# Optical Frequency-Domain Reflectometry Based Distributed Temperature Sensing Using Rayleigh Backscattering Enhanced Fiber

**DOI:** 10.3390/s23125748

**Published:** 2023-06-20

**Authors:** Ziyi Lu, Ting Feng, Fang Li, Xiaotian Steve Yao

**Affiliations:** 1 Photonics Information Innovation Center, College of Physics Science & Technology, Hebei University, Baoding 071002, China; 2 Hebei Provincial Center for Optical Sensing Innovations, Baoding 071002, China

**Keywords:** distributed optical fiber sensing, Rayleigh backscattering enhanced fiber, optical frequency-domain reflectometry, temperature measurement

## Abstract

An innovative optical frequency-domain reflectometry (OFDR)-based distributed temperature sensing method is proposed that utilizes a Rayleigh backscattering enhanced fiber (RBEF) as the sensing medium. The RBEF features randomly high backscattering points; the analysis of the fiber position shift of these points before and after the temperature change along the fiber is achieved using the sliding cross-correlation method. The fiber position and temperature variation can be accurately demodulated by calibrating the mathematical relationship between the high backscattering point position along the RBEF and the temperature variation. Experimental results reveal a linear relationship between temperature variation and the total position displacement of high backscattering points. The temperature sensing sensitivity coefficient is 7.814 μm/(m·°C), with an average relative error temperature measurement of −1.12% and positioning error as low as 0.02 m for the temperature-influenced fiber segment. In the proposed demodulation method, the spatial resolution of temperature sensing is determined by the distribution of high backscattering points. The temperature sensing resolution depends on the spatial resolution of the OFDR system and the length of the temperature-influenced fiber. With an OFDR system spatial resolution of 12.5 μm, the temperature sensing resolution reaches 0.418 °C per meter of RBEF under test.

## 1. Introduction

Fiber optic sensors offer numerous advantages, including high sensitivity, immunity to electromagnetic interference, and adaptability to various environments. These sensors have demonstrated significant application value in aerospace, geological exploration, and large-scale structural health monitoring [1,2,3]. Optical frequency-domain reflectometry (OFDR) is a technique that leverages the inherent characteristics of Rayleigh backscattering (RBS) in optical fibers for distributed sensing [4,5,6]. OFDR has been widely used for distributed sensing and measurement of physical parameters such as strain and temperature, as it has high measurement accuracy and spatial resolution [7,8,9].

The commonly employed parameter demodulation method in OFDR is based on cross-correlation calculations to determine the shift in the RBS spectrum. The distributed RBS results from a weak refractive index modulation distribution, which can be interpreted as distance-resolved random weak fiber Bragg gratings (FBGs) along the fiber axis. External temperature variations induce local refractive index changes in the fiber, leading to shifts in the reflected spectrum of the local weak FBGs. By calculating the offset between the reference RBS and the measurement RBS spectra before and after the temperature change using a cross-correlation algorithm, the temperature change along the fiber can be determined [10]. While this method has good stability and accuracy, it is limited because the temperature measurement accuracy and the measurement spatial resolution cannot be simultaneously improved [11]. Various research groups have addressed this challenge, proposing new data processing algorithms [12,13] or fiber backscattering enhancement mechanisms [14,15] to improve temperature detection sensitivity.

There are currently numerous backscattering enhancement approaches available. One involves increasing the numerical aperture of optical fibers by changing the chemical doping concentration of the fiber core to achieve RBS enhancement [16]. Another approach utilizes the phase mask method with an ultraviolet (UV) laser to create FBG arrays in photo-sensitive fibers [17] or enhances the RBS profile of ordinary single-mode fibers (SMFs) through direct UV laser exposure [18]. RBS enhancement can also be achieved using femtosecond laser direct writing technology, where microstructures created in the fiber core exhibit stable performance at high temperatures [19]. For example, femtosecond laser direct writing of random grating arrays has been reported for OFDR-based distributed temperature fiber sensing with high accuracy [20].

This paper introduces a novel distributed temperature sensing technique based on a newly developed RBS enhanced fiber (RBEF) as the sensing medium. The RBEF utilizes random high RBS peaks along the fiber as the feature points, and a self-constructed OFDR measurement system is employed for sensing. The displacement values of the high RBS peaks before and after temperature changes along the fiber are analyzed using the sliding cross-correlation method. By calibrating the mathematical relationship between displacement changes and temperature variations, the location and magnitude of temperature changes in the fiber can be directly demodulated. Experimental results confirm the linear relationship between temperature change and displacement variation of the high RBS peaks, yielding a sensitivity coefficient of 7.814 μm/(m·°C). Additionally, the spatial resolution of temperature variation is shown to be solely determined by the distribution of high RBS peaks, while the temperature resolution is influenced by the spatial resolution of the OFDR system and the fiber length under test.

## 2. Experimental System and Principles

### 2.1. OFDR System and RBEF

**Figure 1:**
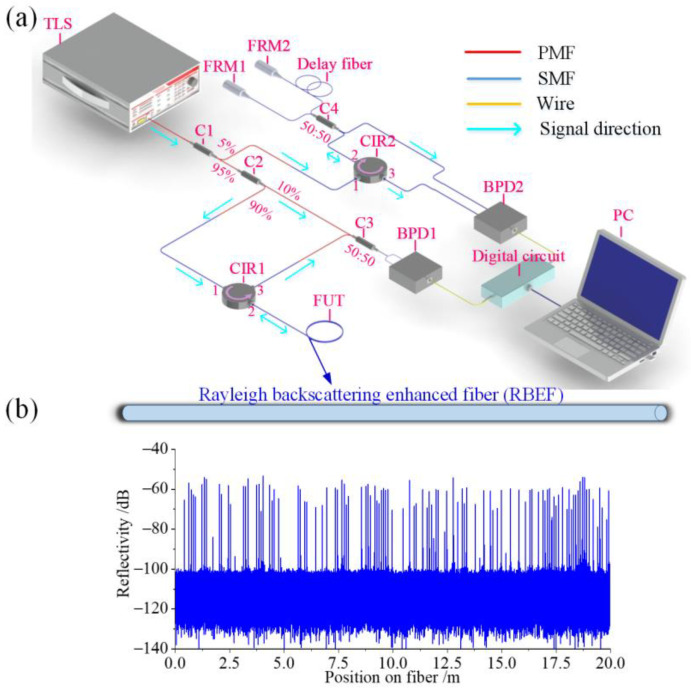
(**a**) OFDR system; (**b**) Distribution of scattering intensity in RBEF measured by OFDR.

Figure 1a shows the configuration of the OFDR system employed in the temperature sensing experiment. The system incorporates a narrow linewidth tunable laser source (TLS, Yenista TUNICS T100S-HP, Lannion, France) with a linewidth, sweep speed, and sweep range of approximately 500 kHz, 2.5 × 10^3^ GHz/s (20 nm/s), and 10^4^ GHz (80 nm, from 1510 nm to 1590 nm), respectively, and an output power of 2 dBm. A coupler C1 (5:95) is used to split the light from the TLS into two paths leading to a main interferometer and a k-clock auxiliary interferometer, respectively. From the total, 95% of the light from C1 enters the main Mach-Zehnder interferometer through a coupler C2 (10:90), and therein 10% of the light is used as a reference light sent to a coupler C3 (50:50). The remaining 90% of the light from C2 first enters the fiber under test (FUT) through port 2 of the circulator, CIR1. The backward RBS light from the FUT enters C3 through port 3 of CIR1 and interferes with the reference light from C2. The resulting interfered light from C3 is then detected and amplified by balanced photodetector 1 (BPD1). The remaining 5% of the light from C1 enters the k-clock auxiliary interferometer, composed of a Michelson interferometer (MI). Two Faraday rotation mirrors (FRM1 and FRM2) suppress polarization fading in the MI. The MI’s output is detected and amplified by balanced photodetector 2 (BPD2). The zero-crossing points of the output signal are converted into pulses in the digital circuit, which act as an external clock to trigger the analog-to-digital converter (ADC) for signal acquisition. This process enables equal-frequency interval sampling of the interference signal received by BPD1 in the digital circuit, thus eliminating nonlinear phase caused by nonlinear tuning of the TLS. Since the TLS has a coherence length of ~600 m, in the k-clock auxiliary interferometer, a delay line of ~250 m-long SMF is used to ensure an OFDR measurement distance of >100 m. The accurate position information of the backward RBS light in the FUT is obtained by applying a fast Fourier transform (FFT) to the resampled interference signal.

The RBEF used in this work was provided by Sentek Instrument Limited Liability Company. SMF jumpers were fusion-spliced at both ends of the RBEF for connection to the OFDR measurement system. The RBS distribution of the entire fiber was measured using the OFDR with a spatial resolution of 12.5 μm, as shown in Figure 1b. The introduction of high RBS points in the fiber yields an overall enhancement of about 40 dB in scattering intensity, and each high RBS peak is different and randomly distributed along the fiber, allowing it to serve as the characteristic peak for distributed temperature sensing.

### 2.2. Temperature Demodulation Principle Based on RBEF

**Figure 2:**
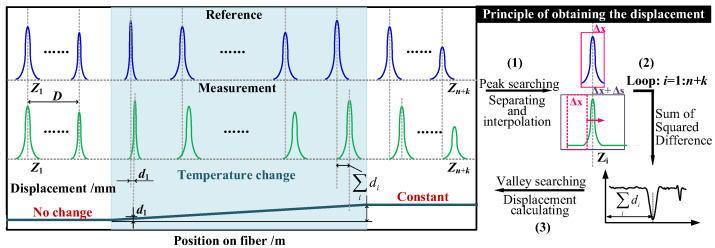
Distributed temperature sensing demodulation principle and procedures using RBEF.

The position-resolved high RBS peaks in the RBEF are easy to identify and can be targeted for the development of demodulation algorithms, as depicted in Figure 2. To establish the correlation between the high RBS peaks and temperature variations, an average distance *D* is defined between adjacent high RBS peaks. Initially, each peak corresponds to a specific position coordinate. Temperature changes then alter the refractive index distribution of the fiber, thereby changing the coordinates of all high RBS peaks in the temperature-affected fiber segment. However, the scattering intensity of each peak and the relative position between peaks remain unchanged.

As shown in the black box on the left side of Figure 2, the first and second rows represent the distribution of reference high RBS peaks (before temperature change) and sensing high RBS peaks (after temperature change) in the distance domain after the FFT operation. Consider an example of *n* + *k* high RBS peaks within a certain length range of the RBEF. Here, *n* represents the number of high RBS peaks within the fiber segment affected by temperature changes, while *k* denotes the number of high RBS peaks located in the succeeding fiber segment unaffected by temperature changes. The temperature change causes a physical displacement of the *i*th (*i* = 1 to *n*) high RBS peak, denoted here as *d_i_*. Preceding the temperature-affected fiber segment, the high RBS peaks show no displacement. However, the coordinates of the high RBS peaks within the fiber segment accumulate, as each peak’s coordinate is affected by the previous displacement of peaks. Specifically, the displacement of the *i*th (*i* = 1 to *n*) high RBS peak is exactly $\sum_{i}d_{i}$. Additionally, the *k* high RBS peaks that follow the temperature-influenced fiber segment do not experience any physical displacement, so their coordinates remain unchanged.

Prior to the data processing illustrated on the right side of Figure 2, two OFDR measurements were conducted to collect the time-domain signals, respectively, before and after the RBEF was subjected to the temperature change. These data were then transformed into the distance domain using the FFT transformation, yielding the RBS intensity distribution relative to position. In Figure 2, step (1) involves the peak searching and data windowing processes. First, peak searching is performed on the reference distance domain data, resulting in the identification of a high RBS peak’s position, *Zi* (*i* = 1:*n* + k), which serves as the center position for moving windows Δx and Δx + Δs. The reference distance domain signal is then intercepted and interpolated using the moving window Δx, while the sensing distance domain signal is intercepted and interpolated using the moving window Δx + Δs. The selection of Δs depends on the length of the temperature-affected fiber. Step (2) entails a cyclic calculation of the correlation coefficient of the intercepted data. The Δx + Δs data is further intercepted using a sliding window of length Δx, and the correlation coefficient is computed by the square difference matching method (i.e., one cross-correlation algorithm) for the two Δx-length signals. A set of correlation coefficients is calculated after *i* sliding loops, and a cross-correlation curve is plotted.

Finally, step (3) involves fitting the cross-correlation curve using the least squares method to determine the valley value, which is then subtracted from the corresponding position of the reference signal to derive the displacement value. Finally, by iterating through all the positions of the high RBS peaks, *Zi*, displacement data can be obtained for the entire sensing fiber. Both the position and magnitude of the temperature change along the entire fiber can be obtained through calibration of the mathematical relationship between RBS peak displacement and temperature change.

It should be noted that in this study, the cross-correlation between the reference and measured signals was limited to a single high RBS peak. Nevertheless, our proposed algorithm facilitates the selection of multiple peaks for demodulation in scenarios where the spacing between adjacent high RBS peaks of a RBEF is exceedingly narrow. However, in cases where the peak spacing is large, selecting a cross-correlation window that encompasses more than one high RBS peak can have a negative effect on both the temperature spatial resolution and the processing speed of the algorithm.

## 3. Experimental Results and Discussion

### 3.1. Calibration of RBEF Distributed Temperature Sensing

**Figure 3:**
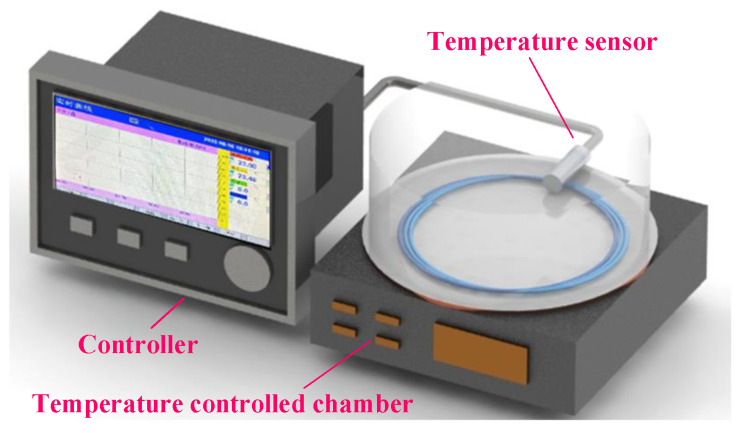
Desk temperature chamber and temperature analyzer for sensing and calibration experiments.

The temperature application and measurement equipment used in the RBEF distributed temperature sensing and calibration experiments are shown in Figure 3. The setup includes a desktop temperature-controlled chamber (General Photonics Cor., STC-101, Chino, CA, USA) and a high-precision thermometer (ConTronix, PT1000, Guangzhou, China) with a temperature sensor. The desktop temperature-controlled chamber itself has high-precision temperature measurement capability, but a thermometer with even greater accuracy (0.01 °C) was used to obtain real-time temperature readings inside the STC-101 chamber for precise, real-time measurements.

The RBEF, serving as the FUT, was connected to the OFDR system. A section of the RBEF spanning from 1.86 m to 11.12 m along the fiber, totaling 9.26 m, as determined using OFDR by setting two markers, was coiled and placed inside the STC-101 chamber set to 20 °C. Simultaneously, the PT1000 temperature sensor was placed in the STC-101 chamber. The temperature of the STC-101 chamber was then incrementally increased from 20 °C to 50 °C in 5 °C steps, with temperature stability being achieved for 10 min at each set temperature before taking measurements. The temperature data recorded by the sensor within the 10-min stabilization period at each temperature setting were averaged to obtain the corresponding actual temperature. The RBS distribution of the RBEF was measured five times at each set temperature, and the average RBS intensity distribution was computed based on the fiber position.

**Figure 4:**
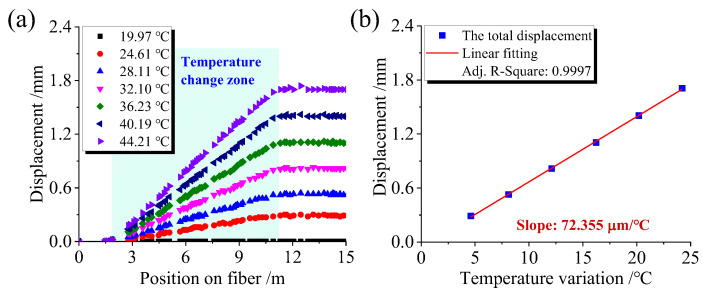
Results of temperature calibration experiments. (**a**) Relationship between displacement of high RBS peaks and corresponding positions along the RBEF under different temperatures; (**b**) Relationship between total displacement of temperature-influenced RBS peaks and temperature variations.

The proposed method was used to successfully demodulate the displacement of the corresponding high RBS characteristic peaks in the fiber segment affected by temperature, as shown in Figure 4a. The horizontal axis represents the fiber position; the vertical axis represents the displacement of high RBS peaks caused by temperature change, $\sum_{i}d_{i}$, as analyzed in Figure 2. Note that in Figure 4, all curves were obtained using the RBS intensity distribution measured at 19.97 °C as the reference signal. Different-shaped points represent measurement data under various temperature values in the temperature-applied fiber segment, where points of the same shape represent the displacement of high RBS peaks resulting from a constant temperature. The displacement of the high RBS peaks in the RBEF part affected by temperature appears to gradually increase, while the displacement of the high RBS peaks after the fiber segment affected by temperature remains constant. By averaging the data in the section where displacement no longer changes, the total displacement of the fiber segment under a specific temperature change can be determined. The relationship between the total displacement of temperature-influenced RBS peaks and temperature variation is shown in Figure 4b.

Linear fitting was applied to the data in Figure 4b, yielding a temperature sensitivity coefficient of 72.355 μm/°C for the temperature-affected fiber segment. Dividing this fitting coefficient by the corresponding fiber segment length (9.26 m) results in a temperature sensitivity coefficient per unit length of 7.814 μm/(m·°C). The theoretical significance of this coefficient is that when the length of the RBEF affected by temperature is 1 m, the total displacement of the high RBS peaks is 7.814 μm for every 1 °C change in temperature.

A precise positioning method is necessary because of the gap between adjacent high RBS peaks (depending on the RBEF fabrication process), which can complicate the accurate determination of the starting and ending positions of the fiber segment affected by temperature change. Theoretically, the position where the displacement of the high RBS peak is zero corresponds to the starting position of the temperature-affected fiber segment, while the position where the total displacement reaches its maximum corresponds to the ending position.

**Figure 5:**
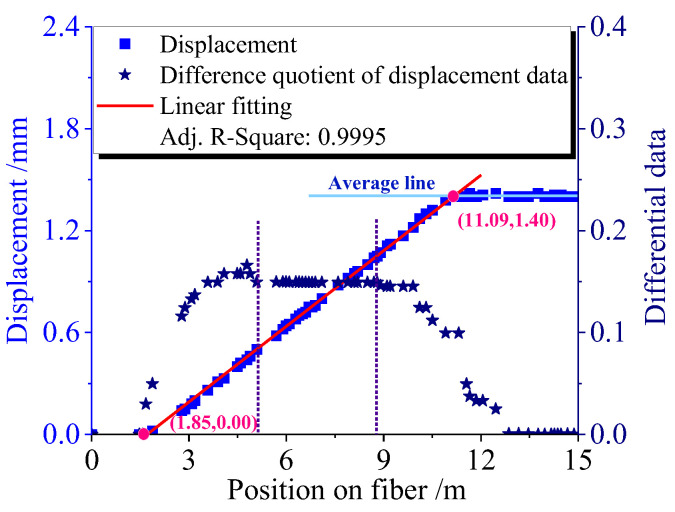
Accurately locating the temperature-influenced fiber segment using the high RBS peak displacement data measured at 40.19 °C in Figure 4a.

Figure 5 illustrates the application of the proposed positioning method using displacement data measured at 40.19 °C (from Figure 4a). The deep blue star points depicted in the figure were determined by implementing a difference quotient algorithm on the displacement data. The differential data (right axis) is able to effectively reflect the changing trend of the displacement data, which enables the determination of the approximate range of fiber length influenced by temperature change. Linear fitting was performed to the partial section of the displacement change data (left axis) corresponding to the constant differential data section between the two dashed lines. The linear fitting yielded an Adj-R. square value of 0.9995, and the fitting line was extended, as marked by the red line in Figure 5. The starting position of the fiber segment affected by temperature change was determined as the position where the vertical axis of the fitting line is zero. Likewise, the ending position is where the fitting line intersects the horizontal line parallel to the *X*-axis, representing the total displacement.

The starting (calculated position 1) and ending (calculated position 2) positions of the fiber segment affected by temperature change, as calculated from the displacement data at various temperatures in Figure 4a, are listed in Table 1. For comparison, the table also provides the starting and ending position data (“measured position 1” and “measured position 2”, respectively) of the fiber segment affected by temperature change obtained by OFDR measurement through the preset markers. As shown in Table 1, when the temperature change is less than 12 °C, the positioning calculation error is relatively large, with a maximum error of 0.13 m. However, when the temperature change is greater than 12 °C, the positioning error is relatively small, with an average error of only 0.02 m.

### 3.2. Temperature Sensing Verification Experiment

**Figure 6:**
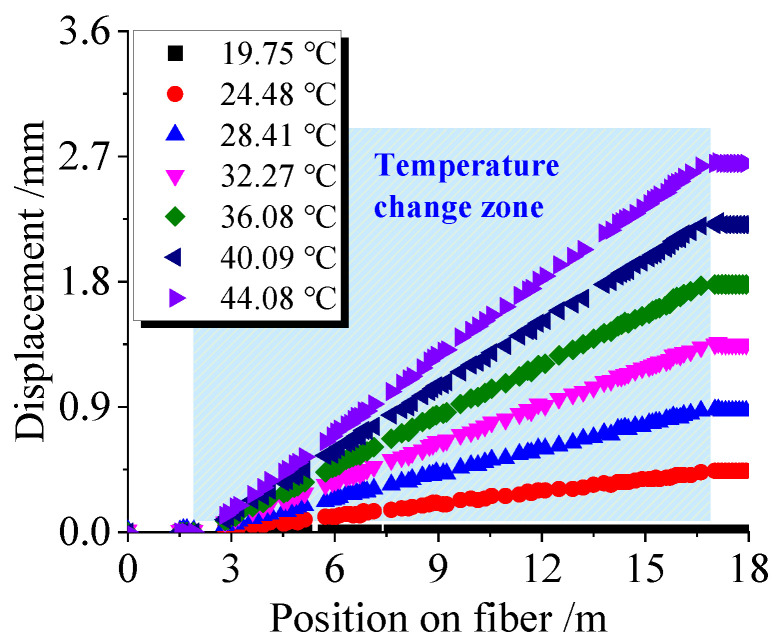
Relationship between displacements and positions of high RBS peaks from the temperature sensing validation experiment.

A temperature sensing verification experiment was conducted to test the accuracy of the temperature calibration coefficient. The same experimental procedure and equipment were used, but the length of the temperature-affected RBEF segment was changed to 14.48 m (between positions 2.22 m and 16.70 m of the fiber). The fiber was subjected to a temperature increase to observe the relationship between the displacement values of high RBS peaks and their positions on the fiber, as shown in Figure 6.

The total displacement of the high RBS peaks at different temperatures was obtained using the demodulation method described above. The temperature change detected by the sensing system was calculated based on the calibrated temperature sensitivity coefficient of 7.814 μm/(m·°C). The temperature value was then demodulated from the sensing experimental data (sensing temperature), as shown in Table 2, based on the initial temperature measured by the PT1000 thermometer (19.75 °C). The temperature value measured by our sensing system was also compared with the temperature value measured by the PT1000 thermometer (measured temperature) (Table 2).

Table 2 shows that the maximum relative error between the measured temperature and the sensing temperature was only −1.84% and the average relative error was −1.12%, which validates the feasibility of the proposed RBEF-based distributed temperature sensing method.

### 3.3. Temperature Sensing Resolution

The measurement resolution of distributed fiber optic sensing technology is determined by the maximum fluctuation amplitude of the tested parameter under steady-state conditions, which is influenced by noise in the instrument and measurements. When the measured signal exceeds the maximum fluctuation amplitude, the sensing system can accurately measure the signal [10].

**Figure 7:**
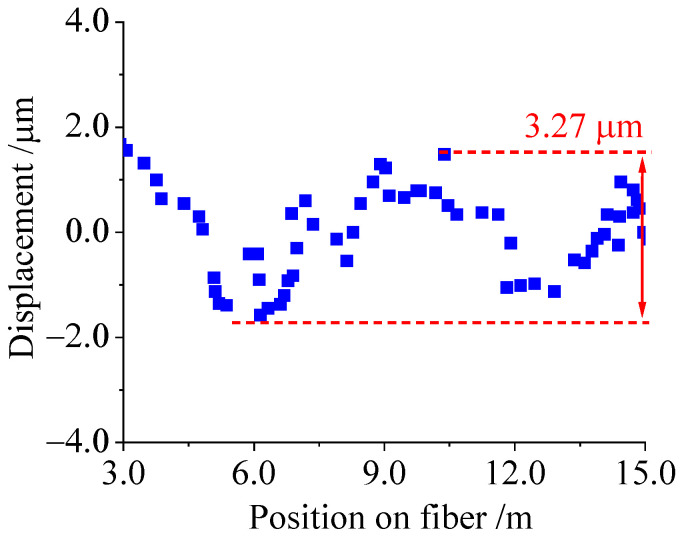
Relationship between displacement of RBS peaks in RBEF and fiber position under constant temperature conditions.

The RBEF (from 3 m to 15 m) was placed in the STC-01 chamber with a preset constant temperature, and the demodulation method (Section 3.2) was employed to measure the displacement change data of the high RBS peaks in the RBEF, as shown in Figure 7. The maximum fluctuation of displacement is 3.27 μm, indicating a maximum uncertainty in the displacement measurement of 3.27 μm. Using the obtained temperature sensitivity coefficient of 7.814 μm/(m·°C), the temperature change per meter of RBEF was calculated to be 0.418 °C/m. This value can (conservatively) be considered the temperature resolution of the sensing system.

Finally, we compared the performance of our approach with the latest studies on distributed temperature sensing, which rely on techniques such as Brillouin optical time-domain reflectometry (BOTDR), Brillouin optical time-domain analysis (BOTDA), Raman optical time-domain reflectometry (ROTDR), and OFDR, as presented in Table 3. Our algorithm, which specifically targets the position of high RBS peaks, results in a significantly reduced computational workload compared to the traditional RBS spectrum cross-correlation algorithm used in OFDR temperature demodulations [21]. The spacing between adjacent high RBS peaks can be adjusted without compromising temperature resolution, which is solely determined by the spatial resolution of the OFDR system. Moreover, the high intensity of the RBS peaks (40 dB higher than that of the RBS distribution in common SMF) enables lower input laser power compared to that required in SMF-based OFDR, or alternatively, a much longer sensing distance in theory. Overall, our proposed approach provides a simple and adaptable method for demodulating distributed temperature sensing.

## 4. Conclusions

In this study, a distributed temperature fiber sensing demodulation algorithm based on OFDR was designed by utilizing the distinctive position-resolved high RBS peaks in the RBEF. Through calibration and verification experiments, a strong linear relationship was established between temperature change and the displacement of backscattering points in the temperature-affected fiber segment. The temperature sensitivity coefficient was determined as 7.814 μm/(m·°C), with an average relative error temperature measurement of −1.12% and a positioning error as low as 0.02 m. The estimated temperature resolution is 0.418 °C/m, derived from the maximum displacement fluctuation of the high RBS peaks under constant temperature conditions.

To further enhance the temperature resolution, optimization of the spatial resolution and noise level of the OFDR system is recommended. Moreover, the temperature spatial resolution appears to be solely influenced by the distribution of high RBS points, which can be improved by modifying the RBEF fabrication parameters. Additionally, the sensing distance of our OFDR system is currently limited to 100 m due to hardware performance constraints. However, this limitation can be addressed by utilizing a TLS with a narrower linewidth and a high-speed data acquisition device. The proposed sensing method offers a straightforward principle, swift data processing, and high efficiency, representing a promising advancement for OFDR-based distributed temperature fiber sensing.

## Figures and Tables

**Table 1:** Comparison between calculated and measured position data of the temperature-influenced fiber segment.

Temperature Variation/°C	Measured Position 1/m	Calculated Position 1/m	Error/m	Measured Position 2/m	Calculated Position 2/m	Error/m
4.61	1.86	1.99	−0.13	11.12	10.99	0.13
8.11		1.89	−0.03		11.01	0.11
12.10		1.79	0.07		11.05	0.07
16.23		1.86	0.00		11.11	0.01
20.19		1.85	0.01		11.09	0.03
24.21		1.85	0.01		11.13	−0.01

**Table 2:** Comparison between temperatures demodulated from sensing experiment data and measured by the PT1000 thermometer.

Measured Temperature/°C	Sensing Temperature/°C	Relative Error/%
24.48	24.03	−1.84
28.41	27.90	−1.80
32.27	32.07	−0.62
36.08	35.88	−0.55
40.09	39.76	−0.82
44.05	43.56	−1.11
Average		−1.12

**Table 3:** Performance comparison between our approach and the latest distributed temperature sensing techniques.

Technique	Temperature Resolution/°C	Positioning Error/m	Sensing Distance/m	Algorithm Efficiency
BOTDR with optical domain demodulation [22]	0.08	0.37	8170	Medium
BOTDA with closed-loop servo control [23]	0.10	2	5000	High
ROTDR with denoising neural network [24]	1.77	1	24,000	Medium
OFDR with polarization-maintaining fiber [25]	0.8	0.013	94	Low
This work	0.418	0.02	100	High

## Data Availability

The data presented in this study are available on request from the corresponding author. The data are not publicly available due to privacy concerns.
